# MiniCD4 protein resistance mutations affect binding to the HIV-1 gp120 CD4 binding site and decrease entry efficiency

**DOI:** 10.1186/1742-4690-9-36

**Published:** 2012-05-02

**Authors:** Katrijn Grupping, Philippe Selhorst, Johan Michiels, Katleen Vereecken, Leo Heyndrickx, Pascal Kessler, Guido Vanham, Loïc Martin, Kevin K Ariën

**Affiliations:** 1 Virology Unit, Department of Biomedical Sciences, Institute of Tropical Medicine of Antwerp, Antwerp, Belgium; 2 Commissariat à l’ Energie Atomique et aux énergies alternatives, Institut de Biologie et Technologies de Saclay, Service d’ Ingénierie Moléculaire des Protéines, Gif sur Yvette, France; 3 Faculty of Pharmaceutical, Veterinary and Biomedical Sciences, University of Antwerp (UA), Antwerp, Belgium

**Keywords:** HIV-1, Resistance, Entry inhibitors, CD4 binding site, Entry efficiency

## Abstract

**Background:**

Binding of the viral envelope protein (Env), and particularly of its gp120 subunit, to the cellular CD4 receptor is the first essential step of the HIV-1 entry process. The CD4 binding site (CD4bs) of gp120, and especially a recessed cavity occupied by the CD4 Phe43 residue, are known to be highly conserved among the different circulating subtypes and therefore constitute particularly interesting targets for vaccine and drug design. The miniCD4 proteins are a promising class of CD4bs inhibitors. Studying virus evolution under pressure of CD4bs inhibitors could provide insight on the gp120-CD4 interaction and viral entry.

**Results:**

The present study reports on the resistance induction of two subtype B HIV-1 against the most active miniCD4, M48U1, and its ancestor, M48, and how these mutated positions affect CD4bs recognition, entry efficiency, and sensitivity to other CD4bs inhibitors. Resistance against M48U1 was always associated with S375R/N substitution in both BaL and SF162; M48 resistance was associated with D474N substitution in SF162 and with H105Y substitution in BaL. In addition, some other mutations at position V255 and G471 were of importance for SF162 resistant viruses. Except for 474, all of these mutated positions are conserved, and introducing them into an SF162 Env expressing infectious molecular clone (pBRNL4.3 SF162) resulted in decreased entry efficiency. Furthermore, resistant mutants showed at least some cross-resistance towards other CD4bs inhibitors, the V3 monoclonal antibody 447-52D and some even against the monoclonal antibody 17b, of which the epitope overlaps the co-receptor binding site.

**Conclusions:**

The mutations H105Y, V255M, S375R/N, G471R/E, and D474N are found to be involved in resistance towards M48 and M48U1. All mutated positions are part of, or in close proximity to, the CD4bs; most are highly conserved, and all have an impact on the entry efficiency, suggesting their importance for optimal virus infectivity.

## Background

The entry process of the Human Immunodeficiency Virus type 1 (HIV-1) into host cells is an important target for the development of preventive vaccines and microbicides. HIV-1 entry is a multi-step process that is mediated by the envelope surface glycoprotein gp120 and the transmembrane glycoprotein gp41 [1,2]. These two subunits constitute a functional heterotrimeric molecule that enables the virus to interact with its primary receptor, CD4 [3-10]. The gp120-CD4 interaction triggers a conformational change that allows binding of gp120 to its co-receptor, most frequently CCR5 or CXCR4, and induces refolding of gp41, finally resulting in fusion with the target cell membrane [11,12].

Three distinct gp120 core structures were revealed: (1) a heavily glycosylated outer domain that is exposed to the surface of the trimer, (2) an inner domain that interacts with the gp41 subunit, and (3) a four-stranded antiparallel β-sheet (*i.e.* the bridging sheet) connecting the outer and inner domains. The CD4bs is formed at the interface of these three domains and buries a large surface of approximately 800 Å^2^. However, the area of actual contact between gp120 and CD4 is much smaller because of cavities formed at the interface. One of these cavities is plugged by the aromatic ring of phenylalanine 43 of the CD4 receptor and, as a consequence, named the Phe43-cavity [11]. This important region, at the interface of the outer and inner domains and the bridging sheet, is well-conserved among the different HIV-1 subtypes and is crucial in the lifecycle of the virus [13].

Because of its high genetic and functional conservation, the CD4bs, and in particular the Phe43-cavity, is considered an extremely interesting target for the development of HIV-1 entry inhibitors [11,13-16].

Several potent CD4bs inhibitors such as soluble CD4 (sCD4), BMS-378806, NBD-556, some llama heavy-chain antibodies (A12, D7, and C8), and various CD4bs antibodies have already been described in literature [17-24]. The best known broad neutralizing monoclonal antibody (mAb) is IgG1b12, which can neutralize 75% of all clade B primary viruses and 40% of all known HIV-1 isolates *in vitro.* It has also been shown to protect macaques from infection [25-29]. Furthermore, recent discoveries have led to some new potent CD4bs mAbs such as HJ16, VRC01, VRC02, VRC03, NIH45-46, 8ANC131, and 12A12 [30-32].

CD4 mimetic compounds, also called miniCD4s, constitute a very promising class of CD4bs inhibitors, e.g. M48 and M48U1 [23,33-38]. Upon binding with HIV-1 and similarly to the cellular CD4 receptor, M48 and M48U1 induce conformational changes in the gp120 architecture thereby exposing masked epitopes on the envelope protein. Furthermore, they were shown to have antiretroviral activities in the nanomolar range [33,35]. Besides their potent antiviral activity, these CD4 mimetic miniproteins also have very interesting physico-chemical characteristics such as their small size (27 amino acids), stable conformation in denaturing conditions such as acidic pH and high temperatures, and relative resistance towards proteolytic degradation [33]. Considering the vaginal environment, it is clear that these characteristics are extremely relevant for microbicide candidates [39]. The most potent miniCD4, M48U1, derived from its ancestor M48, was created by adding a flexible cyclohexylmethoxy group in the para-position of the phenylalanine at position 23 of M48, a residue mimicking Phe43 of CD4. This results in a miniCD4 with high affinity for the conserved and vulnerable Phe43-cavity.

In this study, we investigated the evolution of HIV-1 under miniCD4 pressure to get a better understanding of the miniCD4-virus interaction. To this end, resistance induction in two subtype B viruses was performed; and the genotype, as well as the phenotype, of these viruses was characterized.

## Results

### *In vitro* resistance induction and genotyping

Resistance was induced against M48 and M48U1 by exposing the CCR5-tropic subtype B HIV-1 viruses BaL and SF162 to increasing concentrations of the miniCD4 mimetic proteins M48 or M48U1 in PHA/IL-2 stimulated donor peripheral blood mononuclear cells (PBMCs). In addition, resistance was also induced against an equipotent combination of M48 and M48U1.

In general, resistance was rapidly acquired (see Table 1), which reflects the flexible nature of the envelope glycoprotein and confirms the low genetic barrier for development of resistance towards most entry inhibitors.

**Table 1:** Resistance development in virus isolates exposed to increasing amounts of miniCD4

**Virus**	**miniCD4**	**day of harvest**	**mutations found**	**resistant virus**
BaL	M48	14	none found	
		21	H105H/Y mix	
		28	nd	
		42	H105Y	rM48BaL_a
BaL	M48U1	14	none found	
		21	nd	
		28	nd	
		36	S375R	
		45	S375R	rM48U1BaL_a
BaL	M48U1	14	nd	
		20	S375S/N mix	
		27	nd	
		35	nd	
		48	S375R	rM48U1BaL_b
SF162	M48	14	D474D/N mix	
		21	D474D/N mix	
		28	D474N	
		57	D474N	rM48SF162_a
SF162	M48	13	nd	
		25	nd	
		58	D474N	rM48SF162_b
SF162	M48U1	14	S375S/R mix	
		22	S375R	
		28	nd	
		59	G335R, S375R, G471R	rM48U1SF162_a
SF162	M48U1	14	V255M, L494L/V mix	
		21	V255M, L494V	
		28	V255M, S375N, L494V	
		57	V255M, S375N, L494V	rM48U1SF162_b
SF162	M48 + M48U1	14	D474N	
		21	D474N	
		29	nd	
		50	D474N	rCombiSF162_a
SF162	M48 + M48U1	13	nd	
		25	nd	
		34	D474N, L494L/V	
		58	G471E, D474N, L494V	rCombiSF162_b

Numbering used is according to the HxB2 sequence. nd: not done.

Resistance induction was repeated in two independent experiments (referred to as viruses ‘a and b’). Gp120 sequencing was done at the time when the resistance level was at least 100x above the IC_50_ of M48 or M48U1 and for most virus cultures also at intermediate time points (Table 1). Sequencing indicated that the serine at position 375, part of the constant region 3 of gp120 (C3) and situated in the outer domain, was altered in all M48U1 resistant viruses. An arginine was found in both M48U1 resistant BaL viruses and in one of the M48U1 resistant SF162 viruses (rM48U1SF162_a), whereas an asparagine was observed in the second M48U1SF162 resistant virus (rM48U1SF162_b) (Table 1).

In addition to the S375R/N mutation, both rM48U1SF162 viruses displayed mutations at other amino acid positions. Both G335 and G471 were mutated into an arginine in rM48U1SF162_a after the S375R mutation was induced, whereas V255M and L494V mutations were observed in rM48U1SF162_b prior to the appearance of S375N. The valine at position 255 is, similar to S375, a highly conserved residue lining the Phe43-cavity. Both V255 and G471 are part of C2 and C5, respectively, and contribute to the outer domain, which makes up the largest part of the CD4bs [11,40]. Interestingly, virus rM48U1SF162_b had, besides the S375N and V255M amino acid changes, an additional mutation close to the gp120-gp41 cleavage site; *i.e.* L494V.

**Figure 1:**
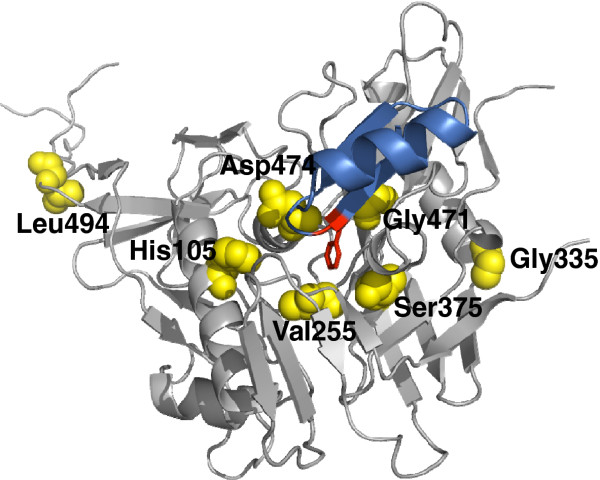
**Locations of gp120 mutations associated with resistance towards miniCD4 proteins M48 and M48U1.** Overall view of gp120 (grey), and M48 (blue). Based on the previously published structures of YU2 core gp120 and Fab 17b in complex with sCD4 or with M48 miniCD4, all the native residues (His105, Val255, Gly335, Ser375, Gly471, Asp474 and Leu494) that are present in BaL or SF162 envelope and were mutated during the resistance induced by M48 and/or M48U1 are represented in yellow and in ball formats. The Phenylalanine at position 23 of M48 is highlighted in red and in stick representation. This drawing was made using PyMOL with coordinates that can be found at pdb accession codes [PDB:2I60] and [PDB:3JWD].

In contrast to the M48U1 resistant viruses, which have most of their mutations in the outer domain, we found that the viruses resistant to M48 and the combination of M48U1/M48 have mutations in the inner domain (Figure 1 and Table 1). Whereas the wild type BaL virus has a histidine at position 105, the rM48BaL virus carries a tyrosine at this position (C1). The SF162 viruses, resistant against M48 (rM48SF162_a and rM48SF162_b) and against the combination of M48U1 and M48 (rCombiSF162_a), displayed the same mutational pattern where the aspartic acid at position 474 (in C5) is altered into an asparagine (D474N). The second SF162 virus resistant to the combination of both miniproteins (rCombiSF162_b) revealed two additional mutations in the outer domain, namely G471E and L494V.

In summary, all mutated positions except two, *i.e.* G335R and L494V are part of or in very close proximity to the CD4bs and particularly near to the Phe43-cavity (Figure 1). The resistance-associated mutations appear often as early as day 14 after the start of drug exposure (Table 1).

Comparative sequence analysis of 3045 HIV-1/SIV Env sequences from the Los Alamos HIV Sequence database (accessed September 2011) showed that most of the mutated positions that we identified are conserved across the most common clades (Table 2). At least 79% of the clade A, B, C and CRF02_AG sequences checked show a histidine at position 105, a valine at position 255, a serine at position 375, a glycine at position 471, and a leucine at position 494. Histidine at position 105 and serine at position 375 are less common in CRF01_AE Env sequences; however, the amino acids found in the M48 and M48U1 resistant viruses at these positions (*i.e.* Y and R, respectively) were not found in CRF01_AE isolates. In general, only 7 (out of 3045 HIV-1/SIV sequences, Table 2) naturally occurring sequences were found carrying the H105Y, V255M, or S375R mutations, suggesting that these mutations are extremely rare. Furthermore, asparagine, glutamic acid, and valine at positions 375, 471, and 494, respectively, were only observed in few cases (between 0% to 8% of sequences, Table 2). In contrast, a glycine to arginine substitution at position 335 and an aspartic acid to asparagine substitution at position 474 appear quite common among the different subtypes (Table 2).

**Table 2:** Prevalence of observed mutations in HIV-1 sequences

				**HIV-1 subtype**			**CRF**	
**Amino Acid Position**			**All**	**A**	**B**	**C**	**01_AE**	**02_AG**
**105**	**H**	wt	83	90	87	97	6	98
	**Y**	mutant	~ 0^a^	0	0	~ 0	0	0
**255**	**V**	wt	96	92	97	98	98	96
	**M**	mutant	0	0	0	0	0	0
**335**	**G**	wt	21	10	14	13	81	11
	**R**	mutant	32	55	46	17	6	39
**375**	**S**	wt	78	91	79	92	1	93
	**R**	mutant	~ 0	~ 0	~ 0	~ 0	0	0
	**N**	mutant	2	0	3	8	0	0
**471**	**G**	wt	78	87	80	80	84	91
	**R**	mutant	~ 0	0	~ 0	0	1	0
	**E**	mutant	4	3	3	6	8	5
**474**	**D**	wt	62	78	68	55	8	95
	**N**	mutant	36	22	31	44	92	5
**494**	**L**	wt	92	92	93	99	99	83
	**V**	mutant	1	1	2	~ 0	0	1

Prevalence (in percentage) of certain amino acids for the most prevalent HIV-1 subtypes at positions mutated in the resistant viruses is given. All: 3045 HIV-1/SIV Env sequences (Los Alamos National Lab data bank 2011); A, B and C correspond to HIV-1 subtypes. Number of Env sequences: subtype A = 186; subtype B = 1001; subtype C = 756; CRF01_AE = 186; CRF02_AG = 100. ^a^ ~ 0 means less than 0.3%.

### Binding experiments with SF162 gp120 mutants

**Figure 2:**
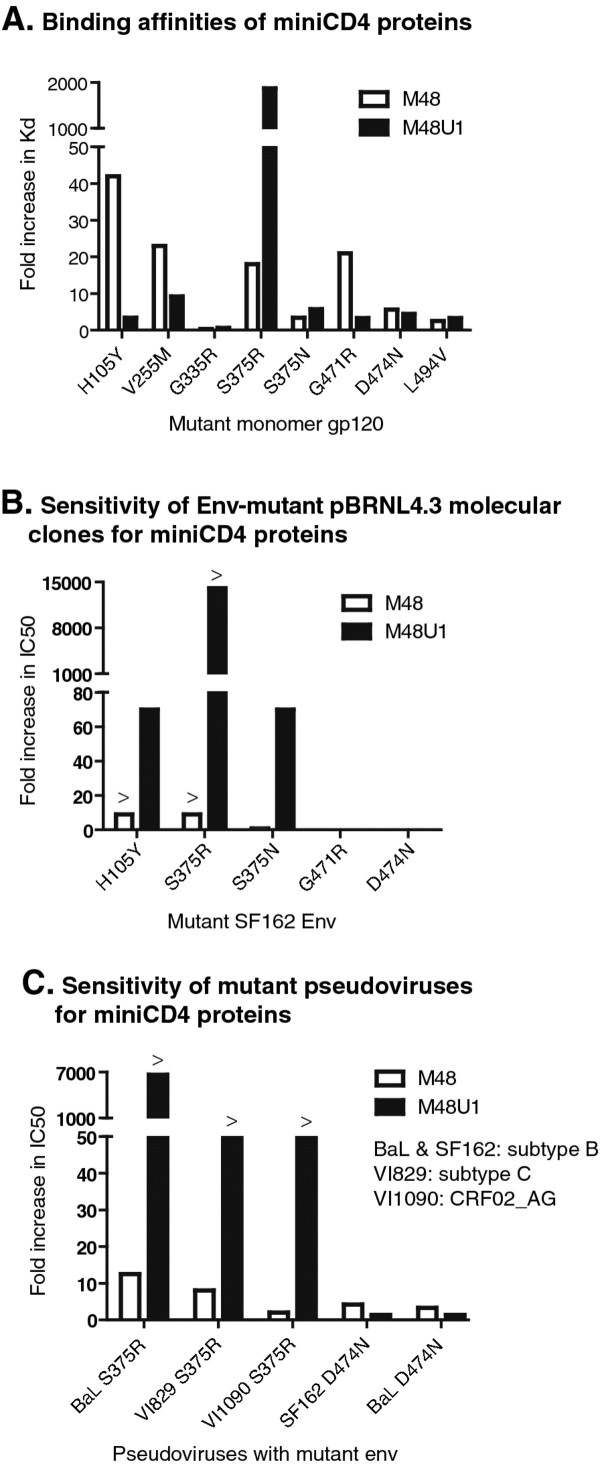
**(A) Binding affinities of fluorescently labelled M48 and M48U1 towards gp120SF162 mutants.** Fluorescently labeled M48 and M48U1 were tested for their affinity towards single site-directed mutants of the SF162 gp120 protein by fluorescence polarization analysis. The fold increase in Kd is plotted relative to the WT SF162 gp120. The H105Y mutation, found in the rM48BaL virus, was also introduced in the SF162 gp120 background. (B) Sensitivity of Env-mutant pBRNL4.3 molecular clones towards miniCD4 proteins. pBRNL4.3 replication competent molecular clones, carrying the H105Y, S375R, S375N, G471R, and D474N mutations were tested for their sensitivity towards the miniCD4 in a TZM-bl based assay. Fold increase in IC_50_ values is given and is calculated as follows: IC_50_ values from the mutant pBRNL4.3 viruses divided by the IC_50_ value from the control wild-type SF162 pBRNL4.3 virus. >; IC_50_ could not be exactly quantified because maximal nontoxic levels of miniCD4 were reached. (C) Sensitivity of mutant pseudoviruses for miniCD4 proteins. The S375R mutation was introduced in the envelopes of the subtype B BaL virus, the primary subtype C VI829 virus, and the primary CRF02_AG VI1090 strain and used to produce mutant pseudoviruses. D474N was introduced in the BaL and SF162 background. Sensitivity towards the miniCD4 was evaluated in a TZM-bl based assay, and results are depicted as fold increase in IC_50_ values, which was calculated as described above. >; IC_50_ could not be exactly quantified because maximal nontoxic levels of miniCD4 were reached.

To explore the contribution of the different amino acid mutations in the interaction between gp120 and the miniCD4 proteins M48 and M48U1, site-directed mutants of SF162 *env* were generated carrying one of the RAMs: H105Y, V255M, G335R, S375R, S375N, G471R, D474N or L494V (see Table 3 for an overview of SDMs). These gp120 mutants were tested in binding assays to assess the interaction with the M48 and M48U1 miniCD4 proteins using fluorescence polarization. Figure 2 shows the fold increase in Kd for the different mutants in comparison with the native SF162 gp120.

**Table 3:** Summary of site-directed mutants

**SF162 gp120**		**Pseudovirus clones**			
Monomer gp120 for binding studies	pBRNL4.3 clones	SF162	BaL	VI829	VI1090
H105Y	H105Y				
V255M	V255M				
G335R					
S375R	S375R		S375R	S375R	S375R
S375N	S375N				
G471R	G471R				
D474N	D474N	D474N	D474N		

Mutations G335R, S375N, D474N and L494V displayed Kd values comparable to SF162 wild type (WT) gp120 and therefore did not have a significant impact on the affinity of HIV-1 Env for both M48 and M48U1, as reflected in a fold Kd close to 1 (Figure 2A). Residues G335 and L494 are located away from the CD4bs (Figure 1) and are accompanied by either S375R/N and/or V255M. Therefore, it is not surprising that they do not influence the affinity for miniCD4 protein binding. We identified D474N as single resistance-associated mutation in the viruses rM48SF162_a, rM48SF162_b, and rCombiSF162_a. D474N is situated at the edge of the inner domain, close to the CD4bs and known to be a direct CD4 and miniCD4 contact residue [36,41]. Nevertheless, loss of the aspartic negative charge does not affect the binding affinity of M48, M48U1 or sCD4 to gp120. This is expected because Asp474 faces the hydrophobic part of miniCD4 *D*-Pro21 and the side chain of CD4 Gln25, and is located too far away for a hydrogen bond.

On the contrary, the mutations H105Y, V255M, S375R, and G471R, all located close to or in the CD4bs (Figure 1), have a clear impact on the Kd values. Envelopes carrying these mutations lost affinity for the M48 miniCD4 protein, whereas only the arginine on position 375 resulted in an extreme reduction in affinity for M48U1, the most potent miniCD4 protein specifically targeting the Phe43-cavity (Figure 2A). Of note, the S375N mutation did not significantly impact the binding affinity.

To further validate these observations, the H105Y, V255M, S375R/N, G471R and D474N mutant SF162 envelopes were also evaluated in the context of replication competent pBRNL4.3 molecular clones (Figure 2B). With the exception of H105Y, results correlated with binding affinity studies and confirmed the importance of V255M and S375R/N in resistance towards M48 and M48U1. Whereas binding of M48 to H105Y mutant monomer gp120 was affected (approx. 40-fold increase in Kd), the impact of this mutation was less apparent in the context of the pBRNL4.3 molecular clone, where gp120 is in its natural trimeric conformation. Similarly, the G471R mutation resulted in small increases in Kd, but had no apparent effect in the context of replication competent HIV. Because the infectivity of the V255M substitution was dramatically reduced, no IC_50_ could be calculated.

Furthermore, S375R and D474N mutations were introduced in different gp120 backgrounds (a primary subtype C strain VI829 and a primary CRF02_AG strain VI1090; Figure 2C), again confirming the pivotal role of the 375 residue in the interaction of M48U1 with envelope. All mutant S375R pseudoviruses had a dramatic increase in IC_50_ for the miniCD4 M48U1 compared to wild type pseudovirus IC_50_ values (Figure 2C).

Of note, although D474N was found as resistance-associated mutation in 4 out of 5 viruses made resistant against M48, this residue did not significantly affect the binding (Figure 2A), nor the sensitivity to inhibition by M48 or M48U1 (Figure 2B and 2C).

### Impact of M48 and M48U1 resistance associated mutants on entry efficiency

The observed impact of some mutations on the binding affinities, together with the conserved nature of most mutated residues, raises the question whether these mutations influence the entry efficiency. To answer this question, mutations H105Y, V255M, S375R, S375N, G471R, and D474N were introduced in the SF162 WT *env* gene using site-directed mutagenesis (Table 3).

About 30% of all sequences have an Arg residue at position G335; and given that the G355R mutant does not impact on the miniCD4’s affinity, this mutant was excluded from further analysis. Also the L494V mutant, although more conserved, but located away from the miniCD4 interaction site and not affecting CD4 binding, was excluded. Although a third of the known gp120 sequences share an Asn474 residue, it was the only mutation found to be associated with M48 resistance in SF162, and therefore it was tested alongside the H105Y, V255M, S375R/N and G471R mutants.

Briefly, mutant envelopes were cloned in a delta-Env pBRNL4.3 molecular clone. Reverse transcriptase (RT) activity of each mutant virus was determined, and equal amounts (40 pg of RT) were used to infect TZM-bl cells. The entry efficiency of each mutant was measured relative to the pBRNL4.3 clone bearing the WT SF162 envelope.

**Figure 3:**
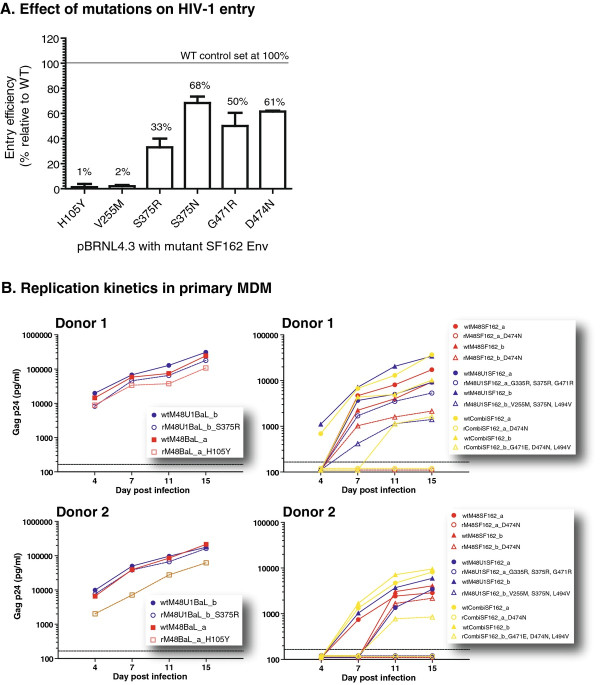
**Evaluation of the effect of the different resistance-associated mutations on HIV-1 entry efficiency and viral growth kinetics.** (A) Mutations H105Y, V255M, S375R/N, G471R and D474N were introduced in the SF162 envelope by site-directed mutagenesis and subsequently cloned into a full –length pBRNL4.3 plasmid, generating replication competent virus. TZM-bl cells were infected with the different viruses using equal amounts of RT activity (40 pg RT). The entry efficiency is expressed as a percentage relative to the pBRNL4.3 bearing the wild type envelope sequence of SF162. The H105Y mutation is related to the rM48BaL virus; however for these experiments this mutation was also introduced in the SF162 envelope. (B) Viral replication of the original resistant SF162 and BaL viruses and their respective control wild type viruses in monocyte-derived macrophages (MDM). Results of two independent experiments are shown. Viral titers were first determined on PHA/IL-2 stimulated PBMCs from each of the two donors. Next, monocytes from the same donor were differentiated into MDM and infected at a multiplicity of infection of 10^-3^. Cultures were incubated for 15 days, and viral growth was determined on different time points by measuring the p24 amount in the supernatants.

Two mutations, H105Y and V255M, had a dramatic negative effect on entry efficiency, resulting in respectively 1% and 2% of infection relative to WT virus (Figure 3A). As mentioned before, these two residues are highly conserved among the most prevalent subtypes (Tyr105 in only 3 out of 3045 sequences and no Met255), are in close proximity to the CD4bs, and substantially affect the binding affinities for M48 (Figure 2A). Nevertheless, the H105Y mutation was only observed in the BaL virus and not in SF162, suggesting that this mutation may be BaL-specific. The molecular clone with an arginine instead of a serine at position 375 had an entry efficiency of only 33% relative to WT virus. The other mutated positions, S375N, G471R, and D474N affected entry efficiency to a lesser extent, with mean percentages of infection relative to the WT clone ranging from 50 to 68%. However, an alanine at position 474 was described previously to make a BaL pseudovirus completely non-infectious, where the same mutation introduced in a YU-2 pseudovirus did not have any effect on the infectivity [42].

Together, these observations suggest that (1) position 474 could be of importance for optimal virus infectivity, and (2) the introduced mutations are context-dependent, *i.e.* the envelop environment is of importance for the phenotype.

### Viral growth kinetics on CD4 negative HOS cells and primary monocyte-derived macrophages (MDM)

Viruses adapted to replicate in CD4 negative cells and in cells expressing low levels of CD4 have been described in literature [43]. Blockage of the CD4 receptor using e.g. CD4 mimetics could skew viruses towards a phenotype of less CD4-dependency, which would theoretically offer the virus the opportunity to replicate in a very diverse subset of cells. To address this, we investigated the viral growth kinetics on a CD4 negative HOS cell line and on primary monocyte-derived macrophages (MDM). MDM express low levels of surface CD4 and represent an *in vivo* target cell population for HIV replication.

All WT and resistant viruses were able to grow in HOS CD4+ CCR5+ cells, whereas none of the viruses replicated in HOS CD4- CCR5+ cells (data not shown). Because MDM are a more representative model, we determined the viral growth in this cell type. MDM were infected with WT and resistant viruses at a multiplicity of infection (MOI) of 10^-3^ and cultures were maintained for 15 days. Viral replication was monitored at different time points using Gag p24 production as a measure of viral growth. Figure 3B shows the results from two independent blood donors. Overall, the WT viruses replicated more efficiently in MDM than the resistant viruses (Figure 3B).

The mutant virus carrying H105Y showed a clear reduction in infectivity (approximately 1 log) in MDM, confirming the low entry efficiency observed in TZM-bl cells. More dramatic effects were seen with virus carrying V255M, S375N and L494V and the virus carrying D474N, with extremely poor replication in MDM.

Together with the observations done in the HOS cells, this finding suggests that resistance induction towards the miniCD4 M48 and M48U1 is not substantially driving the viruses to a CD4 independent phenotype.

### Mapping the sensitivity towards HIV-1 inhibitors and antibodies

**Figure 4:**
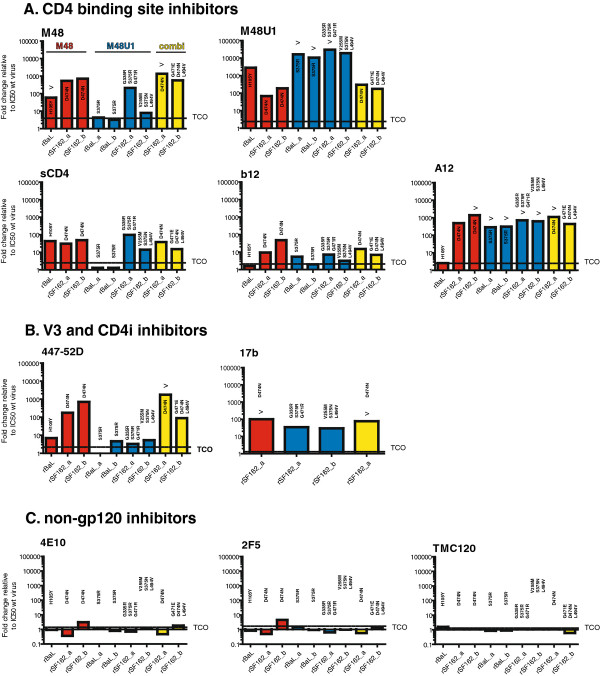
**Mapping the sensitivity of the resistant viruses towards other HIV inhibitors.** The sensitivity against (A) the CD4bs inhibitors M48, M48U1, sCD4, mAb b12, llama nanobody A12, (B) the CD4i mAb 17b, and the V3 mAb 447-52D and against (C) the anti-gp41 mAbs 4E10 and 2F5, and the NNRTI TMC120, were tested in a TZM-bl assay. Fold change in IC_50_ values was calculated as follows: IC_50_ values from the resistant viruses divided by the IC_50_ value from the control wild-type virus. Control viruses are cultured without compound in parallel with the resistance inductions; *i.e.* each resistant virus has its control wild-type virus which was used as a reference to determine ‘wild-type’ IC_50_ values. Technical cut-off (TCO) values were used to define the susceptibility of each virus to a given inhibitor. TCOs were defined as the means and standard deviations (SD) of the IC_50_ values obtained for the control wild-type viruses according to the following formula: TCO = 1 + 2 SD/mean. >; IC_50_ could not be exactly quantified because maximal nontoxic levels of miniCD4 were reached.

To identify the phenotype of the different resistant viruses, we examined potential cross-resistance against various entry inhibitors, including the CD4bs inhibitors s(oluble)CD4, the mAb IgG1b12 and the llama nanobody A12 (Figure 4A), the CD4-induced (CD4i) mAb 17b, and the V3-directed mAb 447-52D (Figure 4B), and the non-gp120 binders 4E10, 2F5 (directed against gp41 MPER) and dapivirine (TMC120; a non-nucleoside reverse transcriptase inhibitor) (Figure 4C) in a TZM-bl assay. The non-gp120 binding agents were included as controls, since they are not expected to be influenced by miniCD4 protein resistance. We used “fold change in resistance” as a measure of cross-resistance, defined as the IC_50_ (50% inhibitory concentration) of the resistant viruses divided by the IC_50_ of the control viruses BaL and SF162, which were cultured in parallel with each resistance induction. As expected, there was no substantial difference in sensitivity of M48 and M48U1 resistant versus wild type viruses (fold change between 0.36 and 4.6) towards the control compounds 4E10, 2F5 and TMC120, confirming that the M48 and M48U1 RAMs in gp120 have no effect on the accessibility of the MPER in gp41, nor on RT function (Figure 4C).

However, cross-resistance towards several CD4bs inhibitors was demonstrated. The virus rM48BaL (H105Y) was not only resistant to M48, but also to M48U1 and sCD4. The other M48 resistant viruses, rM48SF162_a and rM48SF162_b (D474N), showed a different cross-resistance profile. Besides resistance against the two miniproteins and sCD4, a high level of resistance towards the nanobody A12 was seen. Not surprisingly, the two viruses rCombiSF162, possessing the D474N mutation show a similar resistance profile as both rM48SF162 viruses, *i.e.* resistant towards M48, M48U1, sCD4, and A12.

Both rM48U1BaL viruses (S375R) showed extensive cross-resistance to A12, no cross-resistance to M48; and further they were the only mutant viruses which remained completely sensitive to sCD4. The two M48U1 resistant SF162 viruses were cross-resistant to A12, but in contrast to the BaL mutants, displayed also resistance against M48 and sCD4. Finally, SF162 viruses with an asparagine on position 474 presented the highest loss in sensitivity to the mAb b12. Since side chain interactions on this position are involved in b12 binding, this result was not surprising [44].

We also tested whether miniCD4 protein induced resistance affected the sensitivity towards the CD4-induced (CD4i) monoclonal antibody 17b and confirmed that mutant viruses carrying D474N or S375R/N or V255M were resistant to inhibition by 17b (Figure 4B). This antibody recognizes an epitope overlapping the conserved co-receptor binding site, indicating that the changes in the gp120 region documented here for M48 and M48U1 resistance have an impact on the accessibility of the co-receptor binding surface. Furthermore, SF162 viruses resistant towards M48 and the combination of M48 and M48U1, all carrying the D474N mutation, showed substantial cross-resistance to the V3 mAb 447-52D, whereas the viruses resistant towards M48U1 only showed marginal cross-resistance to this mAb (Figure 4B).

Overall, we can conclude that all our mutant viruses showed some cross-resistance to different CD4bs inhibitors, to the CD4i mAb 17b, and to some extent towards the V3-directed mAb 447-52D.

## Discussion

Since many years the CD4bs is considered a very interesting target to block HIV-1 infection. This vulnerable and conserved site on the otherwise genetically diverse envelope protein is essential for the viral entry process. Many studies are being carried out to precisely define the binding surface of CD4 and to pinpoint critical amino acids involved in the gp120-CD4 interaction. So far it is defined as a discontinuous epitope with several distinct regions of gp120 involved [11,45-51]. More than half of the gp120-CD4 interaction surface is formed by the gp120 residues 365–371 and 425–430 as well as the amino acids lining the Phe43-cavity (Trp112, Val255, Thr257, Glu370, Phe382, Tyr384, Trp427, Met475 and the main chains of 256 and 375–377). Although these lining residues contribute little to the direct interaction with CD4, they can certainly have an effect on the gp120-CD4 interaction or on the binding of CD4bs antibodies [11,50]. Most of the interactions between CD4 and the envelop protein are dedicated to the outer domain with the CD4 binding loop as central focus. This loop, formed by ten continuous amino acids (364–373), is essential for the CD4-gp120 binding. Several CD4bs binders, neutralizing and non-neutralizing, have been described to date [26,30-32,47,52,53]. In general, shared regions involved in binding of CD4 and most CD4bs mAbs are the amino acids 257, 368–370, 421–427, and 457. Changes in amino acids Asp368 and Glu370 disrupt the binding of CD4 in CD4bs mAbs and are therefore critical for the CD4bs epitope [11,45-51]

**Figure 5:**
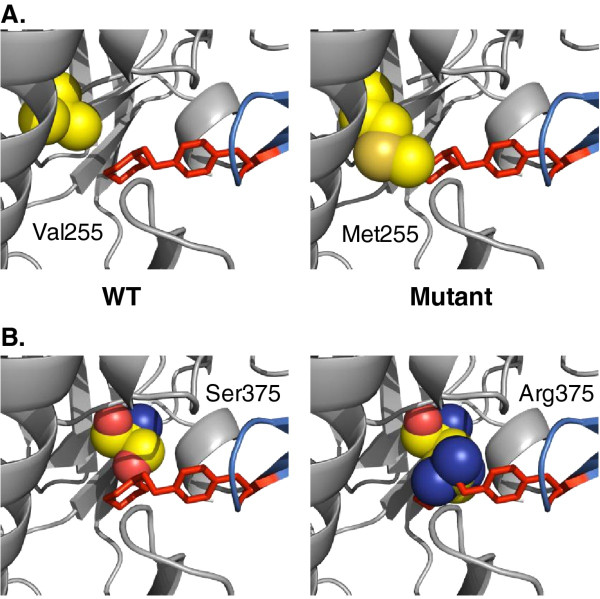
**Close-up views of both V255M and S375R mutants in interaction with M48U1.** (A) Close-up of the Val255 residue in space fill (left) and of the mutant Met255 residue in space fill (right) showing a steric clash with the cyclohexylmethoxy moiety at the para position of phenylalanine 23 of M48U1 in red stick representation. (B) Close-up of the Ser375 residue in space fill (left) and of the mutant Arg375 residue in space fill (right) showing a steric clash with the cyclohexylmethoxy moiety at the para position of phenylalanine 23 of M48U1 in red stick representation. This drawing was made using PyMOL with coordinates that can be found at pdb accession codes [PDB:2I60] and [PDB:3JWD].

Here, we describe mutations found in viruses resistant to the miniCD4 proteins M48 and M48U1, two highly active CD4bs inhibitors. Resistance was induced to evaluate the evolution of the virus under miniCD4 pressure. Overall, most mutations found are situated in the outer domain, which makes up the major part of the CD4bs. Two mutated residues, V255 and S375, both highly conserved, contact the Phe43-cavity and are known to influence the interaction of CD4 or CD4bs antibodies with the envelope protein [11,54-57]. A methionine at position 255, found in one of the SF162 M48U1 resistant viruses, together with a S375N and L494V substitution presumably destabilizes and/or occluded the Phe43-cavity (Figure 5A). A decrease in affinity of M48 towards the SF162 gp120 V255M mutant suggests that the CD4bs region changed to some extent. Previously, a V255E substitution was shown to be responsible for *in vitro* selected sCD4 resistant viruses [54]. The polar amino acid serine at position 375 is mutated in all M48U1 resistant viruses. The arginine side chain at this position is predicted to fill the gp120 Phe43-cavity, the main target of M48U1, implying a steric hindrance to the approach of the cyclohexylmethoxy moiety harbored by the modified Phe23 of M48U1 (Figure 5B). As the ancestor miniCD4 protein M48 is lacking this extra moiety, it does not penetrate as deep into the Phe43-cavity, and therefore attachment of this miniCD4 protein is still possible in the presence of Arg375 (Figure 5B). In concordance, our binding studies revealed a significant decrease in affinity of M48U1, compared to the wild type gp120 affinities, towards the SF162 gp120 S375R mutant, but the same was not observed for M48. In addition, we showed that an asparagine instead of a serine on 375, which does not obstruct the Phe43-cavity, had no dramatic effect on the interaction between both M48U1 and M48 and the mutant gp120. Previously, other groups have reported on the importance of position 375 in interactions of gp120 with CD4 and with CD4bs inhibitors. McKeating *et al.* described a virus where a single S375N substitution conferred the virus resistant to a neutralizing human serum containing CD4bs antibodies and another group reported on this substitution in viruses resistant towards sCD4 and NBD-556, a small molecule that mimics CD4 [54,55]. Next, a tryptophan substitution on 375 fills the Phe43-cavity and forces gp120 into a CD4-bound conformation, which seems to contradict with the observed cross-resistance against 17b in this study [57]. This mutation was also involved in resistance towards some CD4bs compounds from the BMS family of entry inhibitors (BMS-806, #155, and BMS-488043) [54,56,58].

The histidine at position 105, highly conserved and part of the inner domain, was only found mutated in the M48 BaL resistant virus; but nevertheless H105Y conferred resistance, not only to M48, but also to M48U1 and sCD4. However, since this mutation was only found in BaL, it may be strain-specific.

Somewhat controversial data were collected for the D474N substitution. The SF162 viruses resistant towards M48 and one of the SF162 viruses resistant against the combination of M48 and M48U1 (rCombiSF162_a) all selected for this D474N substitution as a single mutation. This mutation was shown to decrease the entry efficiency into TZM-bl cells by 39%, but no significant differences were found in binding affinities for M48 and M48U1 towards the D474N mutant SF162 gp120 protein. Furthermore, the pBRNL4.3 replication competent clone and the mutant pseudoviruses carrying D474N failed to reproduce the resistance observed with the SF162 resistant strains. Moreover, this mutation appears quite common in naturally occurring viruses (Table 2). A previous study reported a D474A mutant with nearly wild type affinity for CD4-Ig, but with a marked decrease in neutralization sensitivity [59]. This mutant was also shown to impair viral fusion and fitness, especially for the BaL strain [42]. However, as observed in gp120 three-dimensional structures, Asp474 makes a strong hydrogen bond with Arg476, which is impossible with an alanine residue at this position. Notably, the D474A mutation was also not detected in naturally occurring viruses (Los Alamos HIV-sequences database). Taken together, we do not have a valuable mechanistic explanation for this D474N resistant mutant to date.

It is important to take into consideration the drawbacks of the different techniques used. First, *in silico* modeling and fluorescence polarization binding studies using monomeric gp120 proteins are not fully representative for the native gp120 and gp41 structure; nor do they model the gp120-gp41 interaction, the interaction of the variable loops, and the interaction between the three units that make up a functional spike completely correct. Secondly, there is considerable evidence suggesting that the genetic environment is of importance for optimal envelope interactions and functioning [60-62]. Expressing Env in a non-isogenic backbone could affect the quaternary structure of the envelope protein and hence its function.

By analyzing 3045 sequences of the Los Alamos Database, we found that most mutated amino acid residues are strongly conserved across HIV-1 clades. This conserved nature of the mutated positions strongly suggests that they are critical for the survival of the virus. So, the next question we wanted to address was if the different mutations had an impact on entry efficiency. Therefore, we infected TZM-bl cells with different pBRNL4.3 viruses containing the envelope from wild type or mutant SF162. We showed that all gp120 mutants tested entered target cells less efficiently compared to WT virus. Two mutations resulted in a severe reduction in entry efficiency, with almost no infection observed for H105Y and V255M. The S375R substitution was responsible for a 67% reduction in entry efficiency. Finally, the effect on entry was less pronounced for the viruses bearing the S375N, G471R, and D474N substitutions. Surprisingly, a D474A substitution was previously shown to have a severe effect on viral infectivity in a BaL pseudovirus, but the same was not true for a YU-2 pseudovirus [42]. Again, these results show the importance of the envelope environment for the phenotype.

Skewing viruses towards a CD4 independent phenotype can be a concern when using CD4 mimics. Therefore, we evaluated the viral growth on CD4 negative HOS cells and on CD4^low^ MDM. There was no viral growth observed on CD4 negative HOS cells, whereas all viruses were able to grow on HOS CD4+ CCR5+ cells. Evaluation of the growth kinetics in MDM revealed that WT viruses were more efficiently replicating in MDMs than the resistant ones. Taken together, there is no evidence that the mutations we have identified as key to the development of resistance against the miniCD4 proteins M48 and M48U1 are rendering the virus less dependent on CD4 for entry.

Finally, we wanted to know if the observed mutations had an impact on the inhibitory potential of other CD4bs inhibitors. Therefore, we tested the resistant viruses towards some other CD4bs inhibitors, the mAbs 4E10, 2F5, 17b, 447-52D and the NNRTI TMC120. Taken together, we observed cross-resistance towards all other CD4bs inhibitors to some extent. All viruses except one (rM48BaL) became cross-resistant towards the nanobody A12. The exact recognition site is not yet revealed, but A12 is considered to target a region within or close to the CD4bs since it competes with CD4 and b12 for gp120 binding [22]. Our results indeed support this prediction. Furthermore, only the BaL viruses resistant towards M48U1 showed wild type levels of sensitivity towards sCD4, consistent with data published about the S375W mutation, while the other viruses showed some cross-resistance. Residues lining the Phe43-cavity, such as V255 and the main chain of S375, are known to possibly impact the binding of CD4 or CD4bs antibodies [11,44,54]. Because glycine at position 471 and aspartic acid at 474 have been described to interact with CD4, b12, and VRC01 [11,44,59], the observed cross-resistance of some viruses was not surprising. Some low level cross-resistance against the mAb b12 was observed for the M48 SF162 resistant viruses and for the viruses resistant towards the combination. All these viruses had the exact same position (Asp474) mutated which could account for the observed resistance.

All SF162 resistant viruses were cross-resistant towards the mAb 17b, which targets a CD4-induced region, overlapping the conserved co-receptor binding site. Also some cross-resistance towards the V3 mAb 447-52D was observed for all SF162 resistant viruses carrying the D474N mutation. These results, together with the decreased affinity for 17b, suggest a more occluded co-receptor region in the resistant viruses.

CD4 mimetics are interesting antiretrovirals, mainly because they target a highly conserved site of the HIV envelope protein and confer broad and extremely potent neutralizing capacity. A point of concern, as with many antivirals targeting the envelope, is the relative ease of resistance acquisition against these molecules. However, changes in the highly conserved CD4bs often come at a cost for the virus. Combining miniCD4s with other entry inhibitors or physically linking CD4 miniproteins with molecules targeting e.g. the CD4i site, may increase the barrier for resistance. These and other strategies are currently under investigation.

## Conclusions

The mutations H105Y, V255M, S375R/N, G471R/E, and D474N are found to be of importance for resistance towards the miniCD4 proteins, M48 and M48U1, in subtype B viruses. All mutated positions are part of or in close proximity to the CD4bs and most are highly conserved. Introduction of these mutations into a pBRNL4.3 chimeric virus carrying the SF162 Env had an effect on the entry efficiency, suggesting that these positions are of importance for optimal virus infectivity. Mutant viruses were not able to replicate in CD4-negative HOS cells but did replicate in MDM, a cellular model of low surface CD4 expression. Finally, cross-resistance towards other CD4bs inhibitors, the mAb 17b, and the mAb 447-52D was observed to varying extents.

## Methods

### Antiretroviral compounds and antibodies

Both CD4 mimetic miniproteins, M48 and M48U1, were designed, synthesized and purified at the Commissariat à l’ Energie Atomique (CEA), Institute of Biology and Technologies of Saclay, Gif sur Yvette, France. The non-nucleoside reverse transcriptase inhibitor (NNRTI) dapivirine (TMC120) was kindly donated by Tibotec BVBA, Mechelen, Belgium; the mAb 17b by Dr James Robinson, Tulane University Medical Center and the llama nanobody A12 by Dr. Theo Verrips, the University of Utrecht, Utrecht, the Netherlands. The mAbs b12, 4E10, and 2F5 were purchased from Polymun Scientific, Vienna, Austria while soluble CD4 (sCD4) was purchased from Progenics Pharmaceuticals, New York, USA. The mAb 447-52D was provided by the NIBSC, Hertfordshire, UK.

### Primary cells and cell lines

The Antwerp Blood Transfusion Centre kindly provided buffy coats from healthy donors. Human peripheral blood mononuclear cells (PBMCs) were isolated using Ficoll density gradient centrifugation. PBMCs were cultured and stimulated for 48 hours in RPMI-1640 medium enriched with 1% L-glutamine, 50 μg/mL gentamicin, 10% heat-inactivated fetal bovine serum (FBS) (Lonza, Verviers, Belgium), and 2 μg/mL phytohemagglutinin (PHA) (Remel, Kent, United Kingdom). After 48 hours, cells were centrifuged and subsequently maintained in RPMI-1640 medium containing 15% FBS, 1% L-glutamine, 50 μg/mL gentamicin, 1 ng/mL IL-2 (Gentaur, Brussel, Belgium), 2 μg/mL polybrene (Sigma-Aldrich, Bornem, Belgium) and 5 μg/mL hydrocortisone (Calbiochem, Leuven, Belgium).

To identify the phenotype of the resistant viruses and their respective control viruses, the adherent CD4 and CCR5 expressing TZM-bl cell line, with a firefly reporter gene under HIV LTR control, was used (NIH AIDS Research and Reference Reagent Program, Germantown, USA). Cells were cultured in Dulbecco’s Minimum Essential Medium (DMEM) (Lonza) containing 1% L-glutamine, 10% heat-inactivated FBS, and 50 μg/mL gentamicin and were incubated by 37°C and 5% CO_2_.

293 T cells, used to produce replicate competent mutants, were cultured in DMEM medium (Sigma-Aldrich) containing 1% L-glutamine, 10% heat-inactivated FBS, and 50 μg/mL gentamicin and were incubated by 37°C and 5% CO_2_.

HOS CD4 R5 and HOS R5 cells (NIBSC, Hertfordshire, UK), used to evaluate CD4-independency, were cultured in DMEM (Lonza) containing 1% L-glutamine, 10% heat-inactivated FBS, 0.1% gentamycine, and 1 μg/ml puromycine dihydrochloride (Sigma-Aldrich).

Monocytes were isolated from PBMC by magnetic isolation using CD14 microbeads (Miltenyi Biotec, Bergisch Gladbach, Germany) according to the manufacturer’s instructions, aliquoted and preserved in CellBanker cryogenic medium (Nippon zeyahu Kogyo, Koriyama, Japan). To differentiate monocytes into monocyte-derived macrophages (MDM), monocytes were incubated for seven days in 10% FCS medium containing 50 ng/mL human macrophage colony-stimulating factor (MCSF) (PeproTech, London, UK), medium was refreshed at day 4.

### *In vitro* resistance induction

Resistance was induced by the serial passage of two CCR5-tropic reference subtype B HIV-1 viruses, BaL and SF162. Four million PHA/IL-2 stimulated PBMCs were infected with the viruses at a multiplicity of infection of 10^-3^ in the presence of 50% inhibitory concentration (IC_50_) of M48 and/or M48U1. As a negative control, similarly infected PBMCs from the same donor were cultured in parallel without compound. Once a week, viral replication was evaluated using an in-house p24 antigen capture ELISA [63] and cultures were refreshed as described by Oliviera *et al.*[64]. Once the preferred resistance level was reached, titration of the resistant viruses and their control viruses was performed on TZM-bl cells. Briefly, 100 μl of TZM-bl cells (1x10^5^ cells/mL) supplemented with 30 μg/mL DEAE dextran and 100 μl of a serial dilution of virus were incubated together for 48 hours in a 96-well tissue culture plate at 37°C and 5% CO_2._ Subsequently, 120μL of supernatants were removed and 75 μl of Steadylite HTS (Perkin Elmer, Life Sciences, Zaventem, Belgium) were added. Next, the luciferase activity (a measure for the amount of infectious virus particles) was measured using a TriStar LB941 luminometer (Berthold Technologies GmbH & Co. KG., Bad Wildbad, Germany) and expressed as relative light units (RLU). Finally, the method of Reed and Muench was used to calculate the tissue culture dose for 50% infectivity (TCID_50_) [65].

### Genotyping of resistant and control viruses

Mutations in the envelope gene associated with resistance to miniCD4 were determined by population sequencing. Viral RNA was extracted from culture supernatant using the QIAamp viral RNA kit (QIAGEN, Venlo, the Netherlands). The one step Expand High Fidelity PCR system (Roche Applied Science, Lewes, UK) was used to reverse transcribe and amplify the HIV-1 *env* sequence. To transcribe viral RNA into cDNA primer Wou29 (5′-TGTAAGTCATTGGTCTTAAAGGTACCTG-3′) was used. First round PCR was performed using Wou 26 (5′-GCATCTCCTATGGCAGGAAGAAG-3′) and Wou29. Nested PCR was performed with primers Wou28_NotI (5′-CCGGCGGCCGCTTTGACCACTTGCCACCCAT-3′) and JFES (5′-CGCTGAATTCAGAGCAGAAGACAGTGGCAATG-3′) as described [66]. Purification of the PCR products was done using the Wizard® SV Gel and PCR Clean-Up Kit (Promega, Leiden, the Netherlands). Next, samples were sent for nucleotide sequencing to the VIB Genetic Service Facility (Wilrijk, Belgium). Primers JFES, ED5 (5′-ATGGGATCAAAGCCTAAAGCCATGTG-3′), MSD_S (5′-AATTGGCTGTGGTATATAAAATTATTCATAAT-3′), Henv6154 (5′-AGAGTGGGGTTAATTTTACACATGG-3′), H1E100 (5′-CGGAATTCAGIACAGTACAATGTACACATGG-3′) and gp41R1 (5′-AACGACAAAGGTGAGTATCCCTGCCTAA-3′) were used. Finally, DNAsis software (Hitachi Software Engineering, Molecular Biology Insights, Colorado, USA) and BioEdit Sequence Alignment Editor (Ibis Therapeutics, CA, USA) were used to analyze the sequences. The residue numbering is based upon that of the prototypic HxBc2 HIV-1 envelope glycoproteins, according to current convention (Korber, B. Numbering positions in HIV relative to HxBc2, Los Alamos National Laboratory, 1998).

### Fluorescence polarization binding experiments

The resistance associated mutations were introduced into recombinant gp120 by site-directed mutagenesis. The mutant proteins were expressed transiently using the Freestyle 293 expression system (Invitrogen, Paisley, UK) and purified by affinity column as previously described [67].

The concentrations of the purified gp120 proteins were then standardized by ELISA using the antibody D7324 (Aalto Bio Reagents). The affinities of all the mutants to both fluorescent labeled M48 and M48U1 were determined by fluorescence polarization as previously described [68] on a LJL Analyst reader (LJL Biosystems, Sunnyvale, CA) and by fitting data with a non-linear regression program (Prism, GraphPad software Inc., San Diego, USA). Then, the fold increases in Kds for the different mutants in comparison with the native SF162 gp120 were calculated.

### Site-directed mutagenesis

Site-directed mutants were produced using the QuickChange Lightning Site-Directed Mutagenesis kit (Stratagene, La Jolla, CA) following the manufacturer’s instructions. The TOPO TA vector containing the gp160 sequence of wild type SF162 served as template and mutagenic primers were used to introduce the resistance associated mutations (RAMs). Following PCR, inserts were sequenced as described above to confirm the desired mutations. Inserts containing the RAMs were cloned in pBRNL4.3 Δ Env and subsequently 293 T cells were transfected using the calcium phosphate transfection method (Promega, Madison, WI) to produce replicate competent RAM mutants. The pcDNA4/TO expression vector containing the gp160 sequence of BaL (subtype B), VI829 (primary C strain) or VI1090 (primary CRF02_AG strain) and the psv7d expression vector containing the gp160 sequence of SF162 (subtype B), served as templates for the design of mutant pseudotyped viruses. Mutagenic primers and the QuickChange Lightning Site-Directed Mutagenesis kit were used to introduce the mutations. Subsequently, 293 T cells were co-transfected with the different mutant env expressing plasmids and the pNL4.3. Luc-R-E backbone using the calcium phosphate transfection method (Promega, Madison, WI) to produce pseudovirions.

### Evaluation of entry efficiency of env-mutant viruses

Mutants were quantified on RT activity using the HS-Lenti RT Activity Kit (Cavidi AB, Uppsala, Sweden) as described in the instructions from the manufacturer. Next, 100 μl of TZM-bl cells (1x10^5^ cells/mL) supplemented with 30 μg/mL DEAE dextran were seeded in a 96-well tissue culture plate and 100 μl of virus dilution, containing a fixed amount of RT activity, were added to the cells. Plates were incubated for 48 hours at 37°C and 5% CO_2_, subsequently luciferase activity was measured as described above.

### Representation of the mutated residues and sequence analysis

PyMOL 1.0 (DeLano Scientific, San Carlos, CA, USA) was used to visualize the location of the resistance associated mutations. In order to show these residues, the coordinates of the pdb accession code [PDB: 3JWD] were used to report the structure of the gp120 core and the CD4 was replaced by the M48 structure found in [PDB: 2I60] based on the gp120 from the subtype B virus YU2 in complex with sCD4 and the mAb 17b. The CD4 miniproteins M48 and M48U1 were manually docked into the structure. To determine the degree of amino acid conservation on the altered positions in the gp120 envelope protein, the Los Alamos HIV Database was used (http://www.hiv.lanl.gov/content/sequence/HIV/mainpage.html).

### Drug sensitivity

The inhibitory activity of the different compounds and antibodies was measured in a TZM-bl assay. Fifty μl of (pseudo)virus solution and 50 μl of a serial dilution of compound or 50 μl medium (negative control) have been pre-incubated for 30 minutes at 37°C, 5% CO_2_. Next, 100μL of TZM-bl cells (at 1x10^5^/mL) supplemented with 30 μg/mL DEAE dextran were added to each well and the 96-well plates have been incubated for 48 hours at 37°C, 5% CO_2_. After incubation, luciferase activity was measured. Finally, the inhibitory activity was calculated in GraphPad Prism 5.03 using non-linear regression (GraphPad Software, San Diego, CA, USA).

Technical cut-off (TCO) values were used to define the susceptibility of each virus to a given inhibitor. TCOs were defined as the means and standard deviations (SD) of the IC_50_ values obtained for the control wild-type viruses BaL and SF162 according to the following formula: TCO = 1 + 2 SD/mean.

### Viral growth on MDM and HOS cells

Stimulated PMBCs were used to titer control wild-type and resistant viruses. Briefly, 100 μl of a serial dilution of virus were added to 100 μl of PBMCs (0.75 x 10^6^/ml) in a 96-well tissue culture plate, which were incubated for 2 h at 37°C, 7% CO_2._ After incubation the inoculum was washed away, and cultures were incubated for 7 days at 37°C, 7% CO_2_. At day 7, p24 was measured using an in-house p24 antigen capture ELISA. The method of Reed and Muench was used to calculate the tissue culture dose for 50% infectivity (TCID_50_) [65]. The monocyte aliquots, coming from the same buffy coat, were thawed and incubated for seven days in 10% FCS medium containing 50 ng/mL human macrophage colony-stimulating factor (MCSF) (PeproTech, London, UK), medium was refreshed at day 4. At the end of the incubation period, macrophages were gently scraped (Greiner Bio-One) from the plate and 150 μl of cells (75 x 10^3^ cells) were seeded in a 96-well tissue culture plate in 10% FCS medium. Subsequently, 50 μl of virus were added at a multiplicity of infection of 10^-3^ and plates were incubated for 24 h. After incubation, plates were washed thoroughly to wash away the inoculum. Next, we harvested supernatants at different time points and measured p24, using our in-house p24 antigen capture ELISA, to evaluate viral growth in the macrophage cultures.

Viral growth on HOS R5 CD4 and HOS R5 cells was determined by titrating viral stocks on these cell lines. Therefore, 100 μl of a serial dilution of the viruses were incubated with 100 μl of cells (0.10 x 10^6^/ml) for 24 h by 37°C, 7% CO_2_. Inoculum was washed away after incubation and cells were incubated for 7 days. Half of the medium was refreshed on day 3 of incubation. At day 7, medium was harvested and viral growth was measured by Gag p24 quantification in the culture supernatant.

## Competing interests

The authors declare that they have no competing interests.

## Authors’ contributions

KG performed the majority of the study design, the experimental work, and the data analysis. PS, LM, LH, KKA contributed to the study design, the experimental work and the data analysis. JM and KV contributed to some experimental work. GV and PK contributed to the study design. KG and KKA drafted the manuscript. All authors were involved in critically revising the manuscript. All authors read and approved the final manuscript.
